# Beyond the 9-to-5 grind: workaholism and its potential influence on human health and disease

**DOI:** 10.3389/fpsyg.2024.1345378

**Published:** 2024-11-06

**Authors:** Shahnaz Aziz, Ciara Covington

**Affiliations:** Department of Psychology, East Carolina University, Greenville, NC, United States

**Keywords:** workaholism, poor health outcomes, biomarkers of cardiovascular diseases, type 2 diabetes, future research directions

## Abstract

Workaholism is often considered a conventional word in the general population to portray those individuals who continuously work and find it challenging not to work. It is usually described as a work addiction and operationalized as a compulsive need to work excessively hard. However, the concept of workaholism remains poorly understood. The first objective of this review is to define workaholism, followed by its related concepts, and how it is assessed. Notably, we distinguish workaholism from work addiction and work engagement. Next, we review the current research literature, largely from the last two decades, to suggest that workaholism contributes toward a wide range of health outcomes, ranging from sleep to stress. In particular, we focus on evidence suggesting that workaholism may be associated with differing risk factors for cardiovascular diseases and potentially other related metabolic abnormalities. Finally, we discuss potential limitations of the existing literature on workaholism, and we provide future directions for this emerging field. Specifically, we underscore the need to link workaholism with more biomarkers of metabolic diseases, such as those related to inflammation, the gut microbiome, and glucose homeostasis. In addition, we highlight the importance of establishing causality between workaholism and poor health outcomes, such as cardiovascular diseases and type 2 diabetes.

## 1 Introduction

Workaholism goes well beyond the number of hours worked, despite the fact it is colloquially used to describe employees who work hard, work many hours, or are obsessed with their work. Recently, Andersen et al. (2023) published the first meta-analysis and systematic review pertaining to the prevalence of workaholism. They determined that the pooled workaholism prevalence was 14.1% (please refer to Andersen et al. for details), thereby enhancing the idea that workaholism is a prevalent issue for employees, organizations, and societies. Workaholism is also a problem that spans across different types of workers or industries; thus, further stressing the importance to examine these issues. Workaholism spans across white-collar and blue-collar workers alike (Haar and Roche, 2013). For instance, in a sample of 100 blue-collar employees from New Zealand working in the construction industry, primarily in skilled (e.g., forklift driving) and manual (e.g., driving) labor, results mirrored other studies which have examined the workaholism triad (i.e., high work involvement, high work drive, and low work enjoyment; McMillian et al., 2002) in white-collar professionals (Haar and Roche, 2013). Blue-collar workers reported comparable harmful influences from the workaholism triad (e.g., work involvement was positively related to self-reports of anxiety, depression, and insomnia), which underscores the notion of workaholism as a universal concept.

Workaholism can negatively affect employers through increased healthcare costs and absenteeism (Falco et al., 2013; Mastudaira et al., 2013). Yet, there is a major gap in the literature on our understanding of how workaholism contributes toward poor health outcomes, particularly those related to specific metabolic diseases. To address this gap, the first objective of this review is to provide a brief history of the conceptualization of workaholism and the current definition of the construct. This is critical to address given that the definition of workaholism is often misinterpreted. Notably, we distinguish workaholism from work addiction and work engagement, as well as provide a brief overview on how workaholism is assessed. Next, we summarize research evidence to suggest that workaholism contributes toward a wide range of negative health outcomes. We assessed the literature from the past two decades, primarily using PsycINFO and PubMed, focused on workaholism and health outcomes. Specifically, we focus on data that implies an association between workaholism and biomarkers of cardiovascular diseases. We also expand the review to suggest there could be some links between workaholism and other metabolic disorders besides cardiovascular diseases. We conclude the review with a discussion of potential limitations in the field and avenues for future research, including the need to establish causality between differing biomarkers of metabolic diseases and workaholism. To our knowledge, this is the first review that has been conducted on workaholism and human health and disease.

## 2 What is workaholism and how is it distinct from work addiction and work engagement?

The term *workaholism* was first portrayed as an irresistible urge to work, derived from an inherent compulsion, which interferes with one’s life outside work (Oates, 1971). Numerous conceptualizations have arisen over the decades, with the common thread among most definitions being a substantial investment in work (Clark et al., 2016). Workaholics attain fulfillment from working, have an inner need to work, and desire the emotional rush that stems from working hard (Balducci et al., 2018). A meta-analytic review of the workaholism literature concluded it is characterized by a craving to work due to internal pressures, having persistent thoughts about work when away from work, and working longer and harder than would be reasonably expected (Clark et al., 2016). As consensus denotes workaholism is operationally defined by a compulsive need (i.e., preoccupation with work; the cognitive component) to work excessively hard (i.e., perpetual work involvement; the behavioral component) (Clark et al., 2016; Schaufeli et al., 2009), this is how it is conceptualized for this review.

Workaholism has also been conceived as an addiction (Oates, 1971), thereby adding to uncertainty with the term *work addiction*. Furthermore, in the scientific literature, it is typically linked to *work engagement*. Accordingly, it is essential to distinguish among these constructs in order to enhance our understanding of workaholism and its effects.

Some researchers have suggested that workaholism is an addiction. For instance, Sussman (2013) and Spagnoli et al. (2020) assert it is a work addiction, thus, they view it as a treatable medical condition that contains six required elements (i.e., salience, mood modification, tolerance, withdrawal, conflict, and relapse; Griffiths, 2005). While it is associated with several health issues (e.g., anxiety symptoms, reduced quality of sleep; Balducci et al., 2018), this alone does not qualify workaholism as a medical condition. Although these two labels are used interchangeably, workaholism is a general term that carries negative implications of heavy work involvement, while work addiction suggests a more specific experience within the physiological and psychological context of addiction (Griffiths, 2005). Morkevičiūtė and Endriulaitienė (2022) resonate with this notion, implying the lines are distorted, however, workaholism is a personal characteristic or behavioral pattern, whereas work addiction is pathological in nature.

Currently, workaholism and work addiction are not in the *Diagnostic and Statistical Manual of Mental Disorders, Fifth Edition (DSM-5),* in which both would be categorized under the classification of repetitive behaviors or behavioral addictions. At present, the DSM-5 excludes them since “there is insufficient peer-reviewed evidence to establish the diagnostic criteria and course descriptions needed to identify these behaviors as mental disorders” (American Psychiatric Association, 2013).

Workaholism is unlike work engagement in which the enjoyment of working, not the compulsion to work, is a strong driving factor (Schaufeli et al., 2006; Shimazu et al., 2015).Typically, work engagement is misperceived with workaholism (Clark et al., 2020; Mazzetti et al., 2014; Shimazu and Schaufeli, 2009), given that some researchers conceptualize them as polar endpoints of a continuum (i.e., positive and negative work commitment; Huml et al., 2020), whereas others (Kim, 2019) argue they both interact directly (i.e., might increase or reduce each other’s influences). More probable, work engagement lies opposite burnout (i.e., happens when personal needs are unmet) on a distinct continuum (Maslach and Leiter, 1997; Shimazu and Schaufeli, 2009; van Beek et al., 2012).

Spence and Robbins (1992) distinguished amongst six worker types who were characterized based on levels of work involvement, work drive, and work enjoyment. One of the types, *enthusiastic workaholics* (i.e., high involvement, drive, and enjoyment), underscored the possible intersection between work engagement and workaholism. Work involvement is analogous to workaholism’s excessive work aspect (i.e., working beyond needs and expectations) and work engagement’s absorption component (i.e., being preoccupied with one’s work; Clark et al., 2020), as each investigates how employees utilize their time (Spence and Robbins, 1992).

Workaholics find it difficult to withdraw from work, as shown by their propensity to commit personal time to work, work beyond external needs or expectations, and continuously think about work (Scott et al., 1997). They might extend activities, constantly appraise the same work, and undertake more tasks than acceptable, hence reducing potential output (Balducci et al., 2021). Alternatively, engaged workers can become so engrossed in their work, that time goes by quickly and they might feel obliged to continue working in an attentive way (e.g., Schaufeli et al., 2002). Yet, they can detach from work and consider other aspects of life, implying undue work is not fundamentally injurious (Van den Broeck et al., 2011). Likewise, van Beek et al. (2011) coined the *engaged workaholic*, who is characterized by working longer than coworkers, being driven by personally significant work activities (i.e., identified extrinsic motivation), and experiencing burnout levels which are between that of engaged workers and workaholics. Engaged workers usually allocate and derive energy via work since it is personally meaningful (Schaufeli et al., 2002), whereas workaholics ponder about work because they cannot stop doing so (e.g., Scott et al., 1997); these experiences may co-occur.

Notably, engaged workers and workaholics can be discerned from each other in that the former experience fewer physical and mental health concerns (Shimazu and Schaufeli, 2009) and encounter less work-life imbalance, compared to the latter (Huml et al., 2020). Work engagement adds value to organizations since employees are more likely to participate in extra-role activities (e.g., organizational citizenship behaviors) and engaged workers show decreased turnover intentions (Borst et al., 2020). Overall, workaholism is harmful at individual, personal, and organizational levels, whereas work engagement has positive outcomes for each. It is beneficial for practitioners to focus on the well-being of their employees (Aziz and Moyer, 2018). It is also valuable to understand both the importance and the detrimental factors of workaholism. We now shift our attention toward how workaholism is assessed, as there are multiple measures of this concept.

## 3 Assessment of workaholism

Several measures of workaholism exist, including Spence and Robbins’s (1992) Workaholism Battery (WorkBAT), Robinson’s (1999) Work Addiction Risk Test (WART), and Schaufeli et al.’s (2009) Dutch Work Addiction Scale (DUWAS)—please refer to Aziz and Moyer (2018) for an in-depth examination of these commonly-used workaholism measures, as well as Goncalves et al. (2023) for a systematic literature review on these workaholism scales. Other notable measures include the 29-item Workaholism Analysis Questionnaire (WAQ; Aziz et al., 2013), a self-report measure scored on a five-point Likert scale ranging from 1 (*strongly disagree*) to 5 (*strongly agree*)—higher scores indicate greater levels of workaholism. It has been psychometrically tested on a heterogeneous working population and demonstrated strong reliability and validity. The WAQ has an internal consistency of *α* = 0.93 (Aziz et al., 2013).

Clark et al. (2020) developed the 16-item Multidimensional Workaholism Scale (MWS) to assess four key components of workaholism (i.e., behavioral, cognitive, emotional, and motivational), with four items in each facet. The behavioral facet is described as excessively working beyond the job’s requirements; the cognitive dimension pertains to constant thoughts about work; the emotional subscale is associated with adverse emotions felt when not working; and the motivational component is portrayed as the internal pressure to work (Clark et al., 2020). Items are rated on a 5-point scale from *never true* (1) to *always true* (5). The MWS augments existing scales by introducing a motivational piece and further distinguishing an emotional component (e.g., like work enjoyment; Spence and Robbins, 1992). Clark et al. (2020) showed sufficient internal consistency for all subscales (0.82 < α < 0.93), and discriminant validity between the MWS and the Utrecht Work Engagement Scale (Schaufeli et al., 2002).

These measures associate and differ from one another in various ways (see Aziz and Moyer, 2018, for a discussion). As detailed in Aziz and Moyer (2018) and Goncalves et al. (2023), due to the varying means to conceptualize workaholism *and* the different subscales to measure it, we concur that it is under the purview of the researcher to determine how to operationally define workaholism and then, accordingly, utilize a measure to assess the construct based on that definition. Additionally, the aforementioned measures have been readapted when examined in various countries and translated in different languages. Hence, it is critical that future researchers intersect the construct of workaholism and establish validity in different industries, cultures, and countries (Goncalves et al., 2023). Although one’s scale of choice is context-dependent and there is no single best measure to assess workaholism, as a short, psychometrically-sound measure, the MWS is well-suited for assessing multiple aspects of workaholism. Psychometrically, the MWS best fits a four-factor structure, is not redundant with other workaholism scales (i.e., DUWAS, WART, and WorkBAT), and adds incremental validity to previous workaholism measures when predicting emotional exhaustion and depressive symptoms (Clark et al., 2020). Additionally, the MWS encapsulates different aspects of workaholism compared to work engagement, thus, it has better construct validity in comparison to other scales given that workaholism and work engagement differ conceptually.

## 4 The impact of workaholism on health outcomes

Many earlier studies have demonstrated the negative influence of workaholism (e.g., Andreassen, 2014; Griffiths, 2011; Shimazu and Schaufeli, 2009). However, this understanding is primarily grounded on *psychological* health outcomes. For instance, workaholism has been linked to *lower life* and *job satisfaction* (Andreassen et al., 2011; Bonebright et al., 2000). In addition, workaholism is associated with *reduced psychological well-being and happiness* (Chamberlin and Zhang, 2009; Del Líbano et al., 2010). Other researchers have found a negative relation between workaholism and self-esteem (Aziz et al., 2018), as well as social relationships (Ng et al., 2007). The extant knowledge on *physical* health outcomes is less understood. In the following paragraphs, we delineate various studies whose findings imply that workaholism may contribute to specific poor health outcomes—we primarily focus on *physical* health outcomes.

There is some evidence to suggest the workaholic lifestyle is related to poor overall health. Based on data from 400 university faculty and staff members, results showed workaholism was positively related to each burnout symptom (i.e., emotional exhaustion, depersonalization, and reduced personal accomplishment) from Maslach’s Burnout Inventory (Moyer et al., 2017). Additionally, in a sample of 671 Danish employees, those with higher workaholism scores had significantly greater mean scores on the Perceived Stress Scale in comparison to those with lower scores (Lichtenstein et al., 2019). Burnout and work stress align with our objectives in that they are markers of interest for examining metabolic diseases; as discussed later, mental health can be deemed a marker in future research.

Additional studies, described below, support that workaholism is linked to poor health outcomes. In a heterogeneous sample of 189 workers, workaholism was positively linked to systolic blood pressure, an objective health measure and physiological outcome of the stress process; gender did not moderate the relationship (Balducci et al., 2018). Workaholism was also related to a greater probability of disabling back pain and sickness absence, especially due to mental health problems, in a sample of 3,899 Japanese workers; variables were assessed with self-report items (Mastudaira et al., 2013). In a sample of 398 employees within a South African engineering organization, workaholism was related to increased self-reported musculoskeletal complaints (Engelbrecht et al., 2019). Moreover, workaholism is associated with self-reported sleep problems (e.g., daytime sleepiness; Spagnoli et al., 2019).

It is hypothesized the poor health of workaholics stems from the fact that they do not give themselves sufficient time to recover from the strenuous effort they expend on a daily basis (Taris et al., 2005). Workaholics often ignore, or do not notice, warning signs such as aches and pains (Vodanovich et al., 2007). The relationship between workaholism and poor health could partially be explained by the fact that workaholics have less time to recuperate from their exorbitant efforts (Taris et al., 2005). Because workaholics work excessively, they have less time to recover from their workday and engage in non-work activities which positively affect physical health, such as exercise and appropriate sleep patterns. Hence, there is emerging research to support the negative influence workaholism can have on overall health (Aziz and Moyer, 2018). For example, Shukri et al. (2016) investigated how work-related factors influence healthy intentions—low-fat consumption, fruit and vegetable consumption, and physical activity; self-report measures were used to collect data. Women had higher intentions to eat low-fat diets than men, but when intentions were influenced by job demands (a strong correlate of workaholism), higher job demands reduced intentions to eat low-fat diets in men and women. Thus, healthy diet intentions were lower when met with adversity related to job demands.

As observed by the studies described here, our understanding of the impact of workaholism on physiological health outcomes might be restricted because many of the health outcomes were measured with self-reports. A more comprehensive discussion of this limitation is provided in Section 5.

### 4.1 Association between workaholism and biomarkers of cardiovascular diseases

As described in the previous paragraphs, there is an association between workaholism and varying aspects of human health ranging from sleep to stress. However, far less is known about the relationship between workaholism and specific diseases. Globally, cardiovascular diseases are the leading cause of death (Di Cesare et al., 2024). Given that individuals expend the majority of their waking hours doing work, it is essential for us to better comprehend the influence of work on cardiovascular diseases. As there is a need for further investigation in this realm, herein, we now focus on the existing evidence suggesting a link between workaholism and cardiovascular diseases. We then hypothesize about the potential relationship between workaholism and other metabolic complications, such as, type 2 diabetes (T2D).

To exemplify, a rigorous study showed overtime work had a negative impact on coronary heart disease (CHD; Virtanen et al., 2010). The influence of overtime work on the incident of CHD was examined longitudinally in a sample of 6,014 full-time British civil servants free from CHD at baseline (1991–1994). They were followed until 2002–2004. The outcome measure was incident fatal CHD, clinically verified incident non-fatal myocardial infarction, or definite angina. It was concluded 3–4 h of overtime work per day was associated with a 1.60-fold higher risk of incident CHD (Virtanen et al., 2010). Interestingly, this result was independent of conventional coronary risk factors. These risk factors included systolic and diastolic blood pressure, serum high density lipoprotein (HDL—typically considered “good” cholesterol) and low density lipoprotein (LDL—typically considered “bad” cholesterol), triglycerides (i.e., circulating fat), smoking status, diabetes, sleep deprivation, psychological distress, work characteristics, and Type A behavior. Although hours worked is not the sole assessor of workaholism (McMillian et al., 2002), these findings shed light on the potential relationship between work and CHD, as well as other metabolic diseases. For example, some of the risk factors for CHD are also risk factors for the development of insulin resistance and T2D. Notably, high LDL, low HDL, and high triglycerides contribute toward poor insulin responsiveness and glucose homeostasis. This is an avenue for future research, as described below.

Catalina-Romero et al. (2013) examined the relationship between work stress, which is strongly tied to workaholism, and abnormalities in circulating lipids. Work stress was significantly associated with dyslipidemia, such as high LDL, and low HDL levels (Catalina-Romero et al., 2013). This is notable as high LDL and low HDL are major risk factors for development of cardiovascular diseases and associated complication of T2D. Relatedly, Mutambudzi and Henkens (2020) found work stress is linked to prevalent chronic health conditions in older adults. In their study, associations were found between chronic health diseases, general work stress, and emotional and physical demands. It is important to note that in the studies by Catalina-Romero and Mutambudzi, the researchers focused on work stress and not workaholism. We focus on workaholism and health outcomes in this review—work stress and workaholism, though correlated, are not the same; thus, this limits our ability to fully establish the link between their findings and the association between workaholism and their outcomes, which warrants further investigation.

In another study, the relationships between workaholism, sleep problems, and various physiological indicators represented in numerous measures of cardiovascular risk (CVR) were examined. This study relied on 537 employees from five Spanish hospitals (Salanova et al., 2016). CVR denotes one’s probability of enduring a heart attack or a stroke in a period of 5 to 10 years. It was assessed by cardiovascular age, Framingham relative risk score, metabolic syndrome, and nine indicators of isolated CVR (e.g., caffeine intake, blood hypertension, alcohol consumption). The results demonstrated workaholism was linked to poor sleep (e.g., sleep tiredness, sleeping fewer hours on weekends and weekdays) and CVR (i.e., higher relative risk scores, greater caffeine and alcohol intake) (Salanova et al., 2016). Moreover, sleep issues mediated the relationship between workaholism and CVR, thus, workaholism may contribute to poor sleep, which increases CVR by deregulating the stress physiology.

In a sample of 266 employees from a medical school in the Southeastern region of the USA, associations between workaholism, exercise, and self-reported illnesses (i.e., heart disease, high cholesterol, T2D, high blood pressure, cancer, kidney disease, and mental illness), after controlling for demographics (i.e., family history, age, hours worked, income, and gender), were investigated (Aziz et al., 2015). Family history was defined as parents, siblings, grandparents, and/or cousins having had the specified illness. Logistic regression analysis showed workaholism was a significant risk factor for illnesses, even after controlling for demographics—a 1-point increase in one’s workaholism score indicated a 2.245 times greater chance of reporting at least one illness. Moreover, exercise mitigated the long-term health risk linked to workaholism, such that each additional 15 min of exercise increased the odds of *not* reporting an illness by 1.06 (Aziz et al., 2015). Similarly, a study based on 194 participants employed in various professional fields (e.g., education, law, medicine) found those who scored higher on the compulsive tendencies and the control facets of a self-report measure of workaholism, were significantly more likely to indicate a family history of both heart disease and hypercholesterolemia (Aziz et al., 2017). Pertinently, work-related psychological stress, a strong correlate of workaholism, is associated with the risk of developing cardiovascular disease and T2D (Krajnak, 2014).

Overall, these results suggest a potential connection between workaholism and impaired human health, particularly related to biomarkers of cardiovascular diseases. Clearly, there is a need for further investigation, as many of the aforementioned studies did not directly assess the onset or progression of cardiovascular disease and failed to assess more biomarkers with workaholism. These biomarkers include inflammatory markers (e.g., C-reactive protein, cytokines such as interleukines), lipid markers (HDL, LDL, triglycerides), and a wide range of other markers that may be of interest, such as those associated with the gut microbiome (which can be pro-inflammatory or anti-inflammatory), adiposity (BMI and other markers of obesity), and mental health.

Among behavioral patterns, as described above, emerging evidence suggests workaholism is a contributing factor in coronary heart disease and stroke (Aziz et al., 2015; Salanova et al., 2016), two diseases that are often macrovascular complications of and/or complicated by prolonged T2D (Khan et al., 2019). However, the role workaholism may play in contributing toward the etiology of T2D is not known. Establishing a relationship between workaholism and specific metabolic outcomes will provide the basis for testing interventions designed to modify workaholic behaviors and its potential impact on insulin resistance (which contributes to more complications such as non-alcoholic fatty liver disease and risk of infections), prediabetes, and ultimately T2D. Given that T2D is a global pandemic (Unnikrishnan et al., 2017), this line of research could have a strong impact.

## 5 Potential limitations of the existing literature on workaholism

In this section, we first discuss limitations within the area of workaholism. The field of workaholism relies heavily on the use of surveys and often there are issues with this approach. Thus, there may be some concerns about using a workaholism survey in that workaholics might be conceived as being unaware of working compulsively and excessively, and the harmful influence it has on mental and physical health. Using a multi-rater approach whereby data is collected from another source to supplement the primary respondent and to verify information, can be particularly essential in such research. To circumvent this concern, Aziz and Zickar (2006) addressed the issue of subjectivity in which a multi-rater approach was utilized to reduce potential precept-percept bias. Specifically, data from a family member, friend, or coworker (referred to as the “acquaintance”) was also collected. The acquaintance rated the participant on relevant variables, including workaholism, and survey responses were linked. Doing so provided a knowledgeable other check of the participant. Intraclass correlations between the participant and the acquaintance were significant, demonstrating the acquaintances verified the participants’ responses (Aziz and Zickar, 2006). Also, there were no mean differences between them, thus, participants had an accurate depiction of themselves. Hence, a comparison of a participant’s workaholism responses with an acquaintance builds the case they are not in denial. Thus, possible lack of self-awareness when using a workaholism survey is ruled out, and the need to add a second source when administering it is negated due to the convergence shown between the sources.

On a related note, adding potential bias to observations is a risk when using self-report surveys, such as those used to assess workaholism. However, it has been advocated that using such methods does not inevitably result in measurement bias (Chan, 2009). They provide information (e.g., internal states like mood) that may not otherwise be discerned and are simple to disseminate. It is also a misconception that relationships between variables used in self-report assessments are more biased than other approaches (Conway and Lance, 2010). The participant’s own perception is what these variables are trying to gauge, thus, using a self-report survey is the most appropriate way to obtain one’s subjective experience of, in the case of this review, workaholism.

Another limitation in the emerging field of workaholism and metabolic diseases is that several health outcomes were assessed with self-reports. Indeed, as most of the studies described in this review utilized self-report survey data, this may also be deemed a potential drawback in the domain of workaholism research. Specifically, as noted previously, several health outcomes were assessed with self-reports (e.g., Engelbrecht et al., 2019; Mastudaira et al., 2013; Spagnoli et al., 2019), which might explain why our knowledge of workaholism’s influence on physiological health outcomes remains limited; utilizing objective assessments of biological measures is recommended in future studies.

As a final limitation, many of the studies in this review were cross-sectional in nature; hence, this can be a methodological limitation in workaholism research. A cross-sectional research design, particularly in conjunction with self-report surveys, may be considered a pitfall despite its popular use for most topics investigated in industrial-organizational psychology research and other disciplines that depend on survey methods (Spector and Pindek, 2016). Most deem longitudinal designs as advantageous because they can establish causality, but this notion may be inflated (Spector, 2019). Cross-sectional designs should primarily be utilized as they are an efficient and inexpensive way to conduct research on organizational constructs and to determine fundamental relationships that are not presently recognized, especially when beginning a new area of investigation (Spector, 2019). This is valuable as a foundation for theory and the target for intervention (e.g., attention may need to be given to workaholism if we want to develop an intervention to reduce it). Once key relations are established, then longitudinal designs can be implemented to help ascertain the influence of workaholism on health consequences over time. This is especially vital due to the emphasis of our review, as well as the fact that cardiovascular diseases can progress over an extended time frame (e.g., Virtanen et al., 2010).

## 6 Directions for future research

The current literature on workaholism, described above, suggests a link between workaholism and poor health outcomes, particularly metabolic diseases. However, there are several directions for future research, as this line of study is generally in its infancy. We emphasize the critical need to further establish associations and causal mechanisms related to specific biomarkers of diseases and workaholism. For instance, work stress, sleep problems, excess caloric intake, and lack of physical activity are key aspects of lifestyle for prevention and/or treatment of cardiovascular diseases and its associated complication of T2D (Anothaisintawee et al., 2016; Guess, 2018; McEwen, 1998; Morelli et al., 2020). Thus, longitudinal studies that address underlying causal mechanisms by which workaholism may contribute toward impaired metabolic outcomes should be initiated. To further exemplify, workaholism could drive stress, which may adversely impact dietary patterns (i.e., consumption of excess snacks and lack of attention toward healthy foods). In turn, one could follow up in future study designs to investigate how workaholism is promoting excess nutrient overload that is a major driver of impaired hepatic glucose production, skeletal muscle insulin resistance, development of systemic inflammation, and destruction of insulin-secreting pancreatic *β*-cells, which all contribute toward the pathogenesis of cardiovascular diseases and T2D (DeFronzo and Tripathy, 2009; Petersen et al., 2017; Ying et al., 2020). A good starting point would be to first establish associations between workaholism and key biological markers associated with the onset and progression of metabolic diseases, followed by studies of causal mechanisms. Some examples of biomarkers of interest include elevated triglycerides, key gut microbes, C-reactive protein (a common inflammatory biomarker, but other inflammatory cytokines and lipids could be used), fasting glucose/insulin, and glucose tolerance.

As yet another example, which may not be mutually exclusive, workaholism might contribute toward a reduction in physical activity through stress which, in turn, is the mechanism by which workaholism is associated with impaired glucose and lipid metabolism that ultimately contributes toward various pathologies, such as cardiovascular diseases and development of T2D. Thus, one avenue for future studies is to understand if modifying workaholic behavior can improve differing risk factors for metabolic diseases, such as hyperglycemia and hyperlipidemia.

Moreover, most studies involving workaholism and health issues utilize self-report survey data (Salanova et al., 2016; Vodanovich et al., 2007); hence, objective assessments of biological measures should be used to investigate the health of workaholics. Key knowledge could be gained from this novel area of research. First, as it is not established as a risk factor in the biomedical literature, we propose it is critical for researchers be informed about workaholism as an additional psychological factor to be considered in the pathogenesis of differing health outcomes, such as cardiovascular diseases, T2D, and other related health complications and diseases. Second, it may set the basis for future longitudinal studies which will establish causality at a mechanistic level between workaholism and differing metabolic diseases such as cardiovascular complications and prediabetes/T2D. Thus, although it is too premature to have an established model on how workaholism contributes toward the onset and progression of specific metabolic diseases, as discussed above, we recommend future studies examine the potential mediating roles of stress, sleep problems, nutrient intake, and total physical activity on the association between workaholism and impaired metabolic outcomes as a starting point.

## 7 Summary

In summary, the field of workaholism and its influence on differing health outcomes is emerging. Notably, workaholism may be a contributing factor toward a wide range of metabolic diseases, such as cardiovascular diseases and T2D, amongst others. The link between workaholism and differing health outcomes needs to be further established with a deeper investigation of various biomarkers of disease and longitudinal studies that aim to determine causality between workaholism and differing biological outcomes. This will require using tools from two very distinct areas of study to be fused, namely, industrial/organizational psychology with biomedical research. Ultimately, this research will impact the prevention and treatment of a wide range of health afflictions that are potentially affected by workaholism.
